# Consensus on the Terms and Procedures for Planning and Reporting a Usability Evaluation of Health-Related Digital Solutions: Delphi Study and a Resulting Checklist

**DOI:** 10.2196/44326

**Published:** 2023-06-06

**Authors:** Ana Isabel Martins, Gonçalo Santinha, Ana Margarida Almeida, Óscar Ribeiro, Telmo Silva, Nelson Rocha, Anabela G Silva

**Affiliations:** 1 Center for Health Technology and Services Research University of Aveiro Aveiro Portugal; 2 Governance, Competitiveness and Public Policies Department of Social, Political and Territorial Sciences University of Aveiro Aveiro Portugal; 3 Digital Media and Interaction Research Centre Department of Communication and Art University of Aveiro Aveiro Portugal; 4 Center for Health Technology and Services Research Department of Education and Psychology University of Aveiro Aveiro Portugal; 5 Institute of Electronics and Informatics Engineering of Aveiro Department of Medical Sciences University of Aveiro Aveiro Portugal; 6 Center for Health Technology and Services Research School of Health Sciences University of Aveiro Aveiro Portugal

**Keywords:** usability evaluation, Delphi study, user-centered design, design, usability, evaluation, process, development, user, digital, efficient, reporting, quality, applicability

## Abstract

**Background:**

Usability evaluation both by experts and target users is an integral part of the process of developing and assessing digital solutions. Usability evaluation improves the probability of having digital solutions that are easier, safer, more efficient, and more pleasant to use. However, despite the widespread recognition of the importance of usability evaluation, there is a lack of research and consensus on related concepts and reporting standards.

**Objective:**

The aim of the study is to generate consensus on terms and procedures that should be considered when planning and reporting a study on a usability evaluation of health-related digital solutions both by users and experts and provide a checklist that can easily be used by researchers when conducting their usability studies.

**Methods:**

A Delphi study with 2 rounds was conducted with a panel of international participants experienced in usability evaluation. In the first round, they were asked to comment on definitions, rate the importance of preidentified methodological procedures using a 9-item Likert scale, and suggest additional procedures. In the second round, experienced participants were asked to reappraise the relevance of each procedure informed by round 1 results. Consensus on the relevance of each item was defined a priori when at least 70% or more experienced participants scored an item 7 to 9 and less than 15% of participants scored the same item 1 to 3.

**Results:**

A total of 30 participants (n=20 females) from 11 different countries entered the Delphi study with a mean age of 37.2 (SD 7.7) years. Agreement was achieved on the definitions for all usability evaluation–related terms proposed (usability assessment moderator, participant, usability evaluation method, usability evaluation technique, tasks, usability evaluation environment, usability evaluator, and domain evaluator). A total of 38 procedures related to usability evaluation planning and reporting were identified across rounds (28 were related to usability evaluation involving users and 10 related to usability evaluation involving experts). Consensus on the relevance was achieved for 23 (82%) of the procedures related to usability evaluation involving users and for 7 (70%) of the usability evaluation procedures involving experts. A checklist was proposed that can guide authors when designing and reporting usability studies.

**Conclusions:**

This study proposes a set of terms and respective definitions as well as a checklist to guide the planning and reporting of usability evaluation studies, constituting an important step toward a more standardized approach in the field of usability evaluation that may contribute to enhancing the quality of planning and reporting usability studies. Future studies can contribute to further validating this study work by refining the definitions, assessing the practical applicability of the checklist, or assessing whether using this checklist results in higher-quality digital solutions.

## Introduction

### Background

Usability evaluation is essential to ensure the adaptation of digital solutions to their users [1,2]. Usability evaluation is defined as the evaluation of the extent to which a product can be used by specified users to achieve specified goals with effectiveness, efficiency, and satisfaction in a specific context of use [3]. When usability evaluation is part of the development process of digital solutions, these are more likely to allow an interaction that is intuitive, efficient, memorable, effective, and pleasant, which is key for user acceptance and digital solutions dissemination [4]. Poor usability impacts the quality of digital solutions and undermines the objective of ensuring that it is suitable for their users [5].

Usability evaluation methods can be based on expert analysis (inspection methods) or on real user data (test and inquiry methods). Within each method, there are numerous evaluation techniques that must be selected according to the characteristics of users and the stage of development of the digital solution to be evaluated [6,7]. In most cases, usability evaluation involves a combination of several techniques [8].

Usability assumes amplified importance when referring to health digital solutions that intend to help preventing, diagnosing, treating, monitoring, or alleviating a disease or injury in human beings [9,10]. The errors and problems related to digital solutions that occur during the process of interaction between users and digital solutions in the real context of use [11,12] could be avoided, at least partially, if a comprehensive usability evaluation performed continuously throughout the design, development, and implementation process of the health-related digital solution had been implemented [13,14]. Usability testing creates opportunities to make digital health solutions easier, safer, more efficient, and pleasant to use [15,16]. These improved interactive qualities benefit not only the user (patient or caregiver) but also the manufacturer and society. Pressing the wrong button, misreading a number, misplacing a component, skipping a step, or overlooking a warning message are examples of potentially catastrophic actions that can be minimized with proper usability evaluation [15]. Another reason to conduct usability tests of health digital solutions is to meet the device regulators’ expectations [15]. There are already a series of regulations, standards, and guides that standardize the evaluation of usability [14,17]. Human factors engineering has been added to regulatory requirements to reduce the number of errors in the use of medical devices, develop intuitive devices, and reduce training costs for both manufacturers and end users [11]. In addition, usability assessment is now integrated into the design and development of health software, and the need to consider the usability of health information technologies is widely accepted [18]. However, despite the widespread recognition of the importance of usability evaluation, there is a lack of research on consensus on related concepts and reporting of usability assessment [19].

Previous studies have highlighted the need to improve the quality of health-related digital solutions. For example, a review of the quality and content of mobile apps to support lifestyle modifications following a stroke has concluded that overall quality was low [20]. A similar conclusion was reported in a study on the quality of smoking cessation apps [21]. Another systematic review on the methodological quality of mobile apps for pain management and assessment concluded that studies fail to report on several important methodological aspects regarding the evaluation of usability, including the use of valid and reliable measurement instruments or the previous experience of the investigator who conducted the evaluation [22]. Similar findings were reported in a recent scoping review aiming to synthesize the characteristics and procedures reported in the existing literature on the usability evaluation of digital solutions relevant to older adults [19]. A few attempts have been made to provide guidance for good evaluation practice in health informatics [23]. However, these guidelines are not specific to usability [23], not for research purposes [24], and lack detail and specification, which might explain why its use is not widespread. An objective and simple tool that clearly identifies what should be considered and how it should be considered when planning and reporting a usability study are needed.

### Objective

A lack of standardized terms and procedures and good practices across the studies on usability evaluation has been identified, such as failure to report on the characteristics of study evaluators and participants, detail the tasks used for usability evaluation, or triangulate methods and techniques in the evaluation of usability. It is unclear whether the lack of information provided in the manuscripts results only from poor reporting as poor reporting may also reflect insufficient planning. Overall, these findings suggest that there is a need for consensus on the planning and reporting of studies on usability evaluation. Furthermore, a systematic review of criteria to assess mobile health apps and respective definitions found great diversity across the literature, further reinforcing the need for consensus, which may help improve the existing tools [25]. Therefore, this study aims to generate consensus on the terms and procedures that should be considered when planning and reporting a study on usability evaluation both by users and experts and provide a checklist that can easily be used by researchers when conducting their usability studies. Consensus on the planning and reporting of usability studies is likely to improve the quality and comparability of usability results across studies and facilitate further research on the impact of usability on the acceptance and use of digital solutions. Conceivably, it might also contribute to increasing the probability that usability issues are detected and corrected before final solutions reach the market. This level of standardization already exists in other health areas. A concrete example of standardization is the CONSORT (Consolidated Standards of Reporting Trials) Statement [26], which is an evidence-based tool that presents a minimum set of recommendations for reporting randomized trials and offers a standard way for authors to prepare reports of trial findings.

## Methods

### Ethical Considerations

This study was conducted according to the ethical principles that have their origin in the Declaration of Helsinki and was approved by the Data Protection Office of the University of Aveiro (nº27). All participants read the participant information sheet outlining the study objectives and procedures and gave informed consent before entering the study.

### Delphi Method

#### Overview

The Delphi method is a structured process that includes several phases and relies on experts to reach a consensus on a specific topic. In a Delphi study, the experts group participates in several rounds and the results of previous rounds are supplied with each new round, so that the experts are able to reconsider their judgments, revising them when appropriate [27,28]. This method was chosen because it is recommended when the aim is to determine consensus for a predefined problem on which there is little information available, and one must rely on the opinion of experts [28]. Furthermore, previous publications from this study authors [19,29] have shown that usability evaluation is a field where there is high heterogeneity in terms of reporting and meaning of concepts that would benefit from contrasting and congregating opinions from experienced individuals and by allowing the possibility of analyzing their answers in light of other experienced individuals’ answers as facilitated by the Delphi method.

#### Delphi Survey Preparation

Two previously conducted scoping reviews on procedures of usability evaluation for digital solutions contributed to the identification of the terms and definitions, as well as a list of items regarding procedures of usability evaluation both with users and experts [19,29] that were sent to participants in round 1 of the Delphi. The terms identified as being used with inconsistent meanings across the studies were usability assessment moderator, participant, usability evaluation method, usability evaluation technique, tasks, usability evaluation environment, usability evaluator, and domain evaluator. Based on the findings of the same review, a definition was proposed for each term. In addition, the reviews allowed the identification of a list of items regarding the procedures of usability evaluation, including 29 items: 6 related to the usability assessment moderator, 6 to the participants, 5 to usability evaluation methods and usability evaluation techniques, 2 related to tasks, 2 related to the usability evaluation environment, 5 related to the usability evaluator and the domain evaluator, and 3 related with the inspection method.

#### Recruitment of Participants for the Delphi

Invitations to enter the Delphi were sent to the Coordinators of the European Projects of the Health and Care Cluster, Horizon 2020 Research and Innovation program (ACTIVAGE [Activating Innovative IoT Smart Living Environments for Ageing Well], ADLIFE [Integrated Personalized Care for Patients With Advanced Chronic Diseases to Improve Health and Quality of Life] Project, FAITH [Federated Artificial Intelligence Solution for Monitoring Mental Health Status After Cancer Treatment], Gatekeeper, InteropEHRate [Interoperable Electronic Health Records at user edge], Pharaon, SMART BEAR, and Smart4Health) who were asked to send the invitation to all partners in the project or to individual participants (Smart and Health Ageing through People Engaging in Supportive Systems [SHAPES]). To enter the Delphi and be considered experienced on usability, at least one of the following criteria had to be met: (1) have 2 publications on usability evaluation, (2) have participated in the evaluation of usability for at least 2 projects, or (3) have designed at least 2 studies on usability. A sample size of at least 20 participants has been suggested as appropriate [27,28,30].

#### Data Collection and Analysis

##### Overview

This Delphi study was organized in 2 rounds and took place between December 2020 and March 2021 and was held on the internet. In both rounds, individual participants (for the SHAPES project as authors are partners in this project) or the coordinators of the European projects (for the remaining projects) were sent an email explaining the study objectives with a link to a survey, which also included the participant information sheet and the informed consent. Participants remained anonymous during the whole study. The anonymity of participants was kept across the 2 rounds.

##### Round 1

The survey for the first round of the Delphi was divided into 2 parts. The first consisted of a list of terms and respective definitions. Experienced participants were asked whether they agreed or disagreed with the terms and definitions, and then to provide a comment or propose alternative definitions. The second part of the survey was on the procedures of usability evaluation. The study participants were asked to rate the importance of each of the procedures for the planning and reporting of a study on usability evaluation using a 9-item Likert scale (1—“item not at all important” to 9— “item very important”) and add any other procedure that, in their opinion, was important and was not already included in the list of procedures provided. Participants were also asked to provide basic demographic information (age, sex, and country of origin) and professional background.

Once the first round of the Delphi was completed, the results were collated. For the terms and definitions, the suggestions given by the experienced participants were aggregated by definition and then analyzed by a panel of 3 members of our team (AIM—a gerontologist and an expert on usability with more than 10 years of experience conducting studies on usability assessment, AGS—a physiotherapist involved in usability for more than 10 years, and NR—an engineer with more than 30 years of experience conducting and leading research on usability) against the following two criteria: (1) the number of participants giving similar suggestions and (2) the level of consensus already achieved on the definition. Based on these criteria and the overall analysis of the suggested changes and commentaries, this panel decided on the final terms and respective definitions. The consensus was defined a priori as having at least 80% agreement [26] for each term and definition. The agreed definitions were then included in round 2. The same panel analyzed the commentaries and suggestions of new items for the list of procedures of usability evaluation using the same 2 criteria and an additional 1 regarding whether suggestions were specific to the planning and reporting of a study on usability evaluation (rather than general suggestions that would apply to any study). The new items that resulted from this analysis were added to the list of procedures and included in the next round. For items already included in round 1, an additional analysis was made; it consisted of the calculation of the number and percentage of ratings attributed to each item grouped from 1 to 3, 4 to 6, and 7 to 9.

##### Round 2

In this round, experienced participants were asked to reappraise the relevance of each procedure. The graphical representation of round 1 results informed the participants and served as a basis for their decision-making on the degree of importance they wanted to assign to each specific item. Care was taken in writing neutral instructions to minimize influencing participants’ responses.

After round 2, the results were analyzed. Consensus on the relevance of each item was defined a priori when at least 70% or more experienced participants scored an item 7 to 9 and less than 15% of participants scored the same item 1 to 3. Consensus on the irrelevance of an item was considered when 70% or more of participants scored the item 1 to 3 and less than 15% of participants scored the item 7 to 9 on the Likert scale [31].

In both rounds, experienced participants were given 3 weeks to complete the survey and were sent 1 to 2 reminders. Participants’ identity was not disclosed at any time.

**Figure 1:**
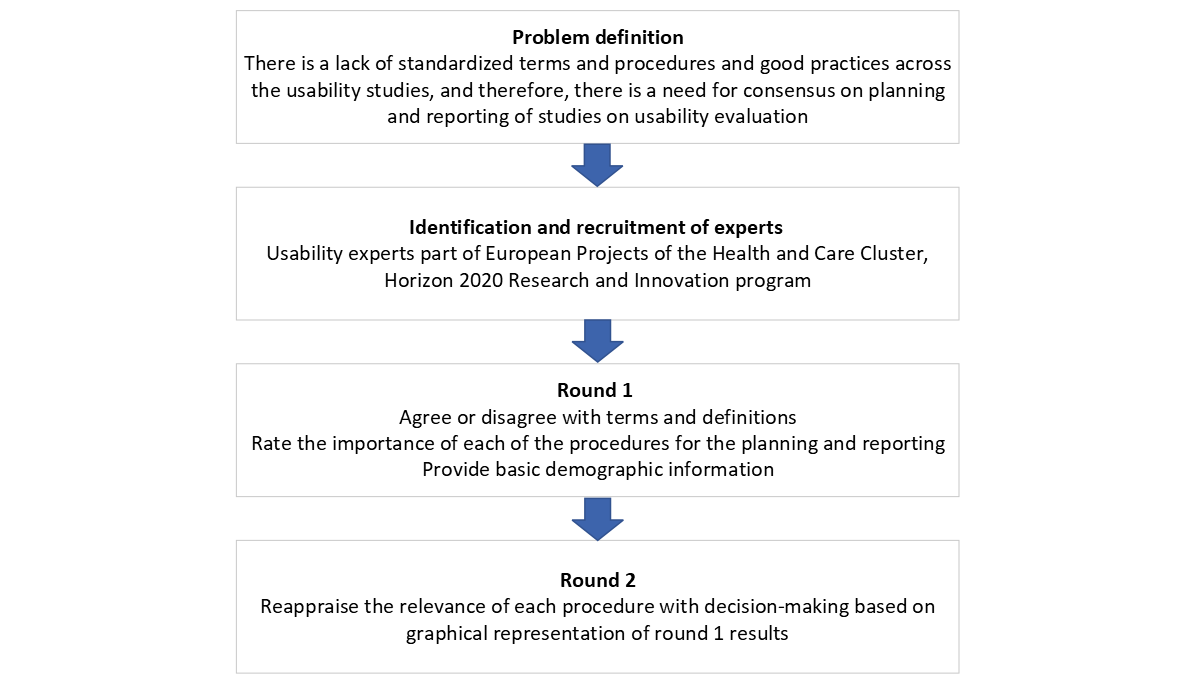
Delphi method diagram.

The diagram represented in Figure 1 summarizes the method implemented for the Delphi study.

## Results

### Participants’ Characteristics

A total of 30 different participants entered the Delphi study: 29 in the first round and 27 in the second round. Participants were from 11 different countries (Table 1), had a mean age of 37.2 (SD 7.7) years, and had experience in designing usability evaluation studies, evaluating usability, or publishing on usability evaluation. Most of them were females (n=20, 67%), with a background related to communication and technology sciences (n=8, 27%) or computer and biomedical engineering (n=12, 40%).

**Table 1:** Participants’ characteristics (N=30).

Characteristics		Value, n (%)
**Gender**		
	Female	20 (67)
	Male	9 (30)
	Prefer not to disclose	1 (3)
**Background**		
	Health and related areas (eg, eHealth researcher, rehabilitation specialist, medical device specialist, and gerontologist)	5 (17)
	Social sciences (eg, psychologist, accessibility specialist, and learning technologies specialist)	5 (17)
	Communication and technology sciences (eg, usability researcher, technology manager, and assistive technology developer)	8 (27)
	Computer and biomedical engineering (eg, computer science researcher, software developer, bioengineer, and robotics engineer)	12 (40)
**Country**		
	Portugal	10 (33)
	Spain	5(17)
	Italy	4 (13)
	Greece	3 (10)
	Germany	2 (7)
	France	1 (3)
	Belgium	1 (3)
	Switzerland	1 (3)
	England	1 (3)
	Netherlands	1 (3)
	Norway	1 (3)
**Experience with usability evaluation**		
	At least designed 2 studies + participated in the evaluation of usability for at least 2 projects and had 2 usability evaluation publications	8 (27)
	At least designed 2 studies and participated in the evaluation of usability for at least 2 projects	6 (20)
	At least 2 usability evaluation publications	3 (10)
	At least participated in the evaluation of usability for at least 2 projects	13 (43)

### Delphi Rounds

#### Round 1—Agreement on Terms and Definitions

The agreement on the terms and respective definitions varied between a minimum of 82.8% (n=24) for the definition of “usability assessment moderator” and 100% (n=29) for the definition of “tasks.” As the predefined minimum agreement rate of 80% was achieved, definitions were not included in round 2 of the Delphi. Nevertheless, 4 definitions were amended following experienced participants’ comments and suggestions (Table 2).

**Table 2:** Results of the agreement and changes made to definitions following round 1 of the Delphi (N=29).

Term	Proposed definition	Agreement, n (%)	Final definition
Usability assessment moderator	The person who conducts the usability evaluation interacts with the participant and guides the session.	24 (83)	The same as the initially proposed
Participant	The person who is asked to evaluate the usability of a product or service and who completes the tasks, following the indications of the evaluator.	27 (93)	The same as the initially proposed
Usability evaluation method	A set of techniques used to perform usability evaluation at different stages of the product or service development (eg, inquiry^a^ or test^b^ methods).	25 (86)	The same as the initially proposed
Usability evaluation technique	A set of procedures used to perform the usability evaluation and collect data of a certain type (eg, brainstorming, questionnaire, and think aloud).	25 (86)	A set of procedures used to perform the usability evaluation and collect either qualitative or quantitative data (eg, focus group, survey, think aloud, and performance)
Tasks	The activities that participants are asked to perform when evaluating the usability of a product or service.	29 (100)	Self-contained or independent activities that participants are asked to perform when evaluating the usability of a product or service within a limited period
Usability evaluation environment	The environment where the evaluation of usability takes place: (1) laboratory or controlled conditions and (2) in a real context, that is, the usability evaluation is carried out in the same context and circumstances where the end product is expected to be used.	27 (93)	The same as the initially proposed
Usability evaluator	A person with knowledge and experience on HCI^c^, usability, and user experience who conducts the usability inspection (eg, an HCI specialist assigned to evaluate the interface of technology for patients with diabetes).	29 (100)	The same as the initially proposed
Domain evaluator	A person with knowledge on the application area of the technology under development (eg, a physician involved in treating patients with diabetes).	28 (97)	A person with knowledge of the application area of the technology under development that provides feedback about functionalities of the technological product or service (eg, a physician involved in treating patients with diabetes who provides feedback about technology for patients with diabetes)

^a^Inquiry methods involve collecting qualitative data from users.

^b^Testing methods involve observing the user while interacting with the product or service and consist of collecting mostly quantitative data.

^c^HCI: human-computer interaction.

#### Round 2—Procedures for Usability Evaluation

In round 1, experienced participants suggested a total of 24 additional items for usability evaluation involving users. Of these, 6 were considered to be repeated items (eg, “have experience with usability methods and techniques,” or “know the methods well and be comfortable in applying the techniques”), 6 were not specific to usability evaluation (eg, “follow ethical guidelines and comply with data protection recommendations” or “create consent forms for the participants”), and another 5 were out of scope (eg, “stress the relevance of the stakeholders and decision makers standpoints” or “detect and match the participants with appropriate user personas”) and were not considered. Therefore, 7 new items were added to the initial list of items and submitted for round 2. In addition, 2 of the items included in round 1 were amended (Table 3). A total of 28 items on procedures of usability evaluation with users were included in round 2.

For procedures of usability evaluation regarding experts, 5 new items resulted from the participants’ suggestions in round 1. Of these, 1 was repeated (“Always use a combination of experts from different domains”) and 2 were not specific for usability planning or reporting (eg, the session should be structured in such a way that avoids bias), hence were not considered, resulting in the inclusion of 2 items in round 2 (Table 3). In addition, the wording of 1 item from round 1 was rephrased for clarification. A total of 10 items on usability evaluation with experts were included in round 2.

Consensus on relevance was reached for 23 (82%) of the 28 items on procedures of usability evaluation with users (Table 4). For the remaining 5 items, consensus was not achieved neither on its relevance nor on its irrelevance..

Consensus on relevance was reached for 7 (70%) of the 10 items on procedures of usability evaluation with experts (Table 5). For the remaining 3 items consensus was not achieved neither on its relevance nor on its irrelevance.

**Table 3:** Results from experienced participants’ suggestions on the procedures for usability evaluation involving users.

Usability evaluation			Items that were rephrased, n		New items proposed by experienced participants, n		Items excluded and reason, n		New items included in round 2, n
**Usability evaluation involving users**									
	Usability assessment moderator	1		4		1^a^ 2^b^		1	
	Participants	0		8		1^a^ 3^b^ 3^c^		1	
	Usability evaluation methods and usability evaluation techniques	1		3		1^a^ 1^b^		1	
	Tasks	0		7		2^a^ 2^c^		3	
	Usability evaluation environment	0		2		1^a^		1	
**Usability evaluation involving experts**									
	Usability evaluator and domain evaluator	0		3		1^a^ 1^b^		1	
	Inspection method	1		2		1^b^		1	

^a^Items excluded as they were repeated.

^b^Items excluded as they were not specific for usability planning and reporting.

^c^Items excluded as they were out of scope.

**Table 4:** Agreement on the items for usability evaluation involving users (N=27).

Planning and reporting procedures for usability evaluation with users			Items grouped from 1 to 3, n (%)		Items grouped from 4 to 6, n (%)		Items grouped from 7 to 9, n (%)
**Usability assessment moderator**							
	Determine the number of usability assessment moderators.	1 (4)		13 (48)		13 (48)	
	Provide the rationale used to establish the number of usability assessment moderators.	2 (7)		10 (37)		15 (56)	
	Specify as inclusion criteria having previous experience with usability evaluation with users or consider adequate training and provide details of the training plan.	0 (0)		2 (7)		*25 (93)* ^a^	
	Detail inclusion and exclusion criteria other than previous experience (eg, academic background or age).	0 (0)		9 (33)		18 (67)	
	Specify whether the usability assessment moderators are external to the service or product development team.	0 (0)		4 (15)		*13 (85)*	
	Specify if observers are included, define their responsibilities, and collect their characteristics (eg, gender, academic background, and previous experience in usability evaluation).^b^	0 (0)		7 (26)		*20 (74)*	
	Detail the usability assessment moderators’ characteristics that should be collected (eg, gender, academic background, and previous experience conducting usability evaluation).^c^	0 (0)		9 (33)		18 (67)	
**Participants**							
	Determine sample size (ie, the total number of participants involved in the evaluation).	0		3 (11)		*24 (89)*	
	Provide a rationale to establish the sample size.	0		6 (22)		*21 (78)*	
	Provide clear inclusion and exclusion criteria (eg, profile definition including age, gender, educational level, digital literacy, and previous experience using the product or service being evaluated).	0		1 (4)		*26 (96)*	
	Provide sampling methods (eg, random, systematic, cluster, convenience, and snowball).	0		8 (30)		*19 (70)*	
	Indicate the setting of participants’ recruitment (eg, community and hospital).	0		4 (15)		*23 (85)*	
	Detail clinical conditions (if relevant for the study) (eg, asymptomatic or with a specific clinical condition or from a specific group—occupational group, the severity of the clinical condition, disabilities, cognitive impairment).	0		2 (7)		*25 (93)*	
	Detail the participant’s characteristics that should be collected (such as age, gender, educational level, and digital literacy).^c^	0		0		*27 (100)*	
**Usability evaluation method and usability evaluation technique**							
	Specify whether a combination of usability evaluation methods was used (eg, using both inquiry and test methods).	2 (7)		4 (15)		*21 (78)*	
	Specify whether a combination of usability evaluation techniques was used (eg, for the inquiry method, combine the questionnaire and interview techniques).	1 (4)		4 (15)		*22 (82)*	
	Provide the rationale for the choice of usability evaluation methods and techniques.	0		5 (19)		*22 (82)*	
	Describe the usability evaluation methods and techniques used and how they are implemented.^b^	0		2 (7)		*25 (93)*	
	When using measuring instruments such as scales or questionnaires, give indicators of their validity and reliability.	0		2 (7)		*25 (93)*	
	Describe the data analysis plan for both quantitative and qualitative data.^c^	0		4 (15)		*23 (85)*	
**Tasks**							
	Provide a detailed description of tasks or present the session script.	0		1 (4)		*26 (96)*	
	Indicate the total number of tasks.	0		6 (22)		*21 (78)*	
	Detail the task-related outcomes and how they are measured (eg, task completion and duration and number of errors).^c^	0		5 (19)		*22 (82)*	
	Detail the conditions for carrying out the tasks (eg, with or without supervision, individually or in the group, with or without a period for familiarization with the digital product or service).^c^	0		0		*27 (100)*	
	Detail the instructions to participants and the way they are presented (eg, verbally, written, and both) and registered (eg, audio, video, screen recorder, and notes from an observer).^c^	0		2 (7)		*27 (100)*	
**Usability evaluation environment**							
	Justify the choice of the usability evaluation environment (eg, lab or field test and remote or face-to-face test).	0		8 (30)		*19 (70)*	
	Specify usability evaluation environment requirements (eg, recording equipment or observer room availability).	0		5 (19)		*22 (82)*	
	Detail the procedures to make the usability evaluation environment safe and comfortable for the participants.^c^	0		9 (33)		18 (67)	

^a^Values in italics denote the items that reached consensus on inclusion.

^b^Items that were rephrased.

^c^New items that emerged from round 1.

**Table 5:** Agreement on items for usability evaluation involving experts (N=27).

Planning and reporting procedures for usability evaluation with experts			Items grouped from 1 to 3, n (%)		Items grouped from 4 to 6, n (%)		Items grouped from 7 to 9, n (%)
**Usability evaluator and the domain evaluator**							
	Determine the number of evaluators involved in the evaluation.	0 (0)		10 (37)		17 (63)	
	Provide the rationale to establish the number of evaluators.	1 (4)		14 (52)		12 (44)	
	Define as inclusion criteria having previous experience in inspection usability evaluation or consider adequate training and provide details of training.	0 (0)		5 (19)		*22 (82)* ^a^	
	State whether the evaluators are external to the product or service development team.	1 (4)		11 (41)		15 (56)	
	Specify whether a combination of evaluators from different domains was used (eg, for a health-related digital service, use both usability and health domain evaluators).	0 (0)		3 (11)		*24 (89)*	
	Provide clear inclusion and exclusion criteria.^b^	1 (4)		1 (4)		*25 (93)*	
**Inspection method**							
	Detail the protocol to conduct the inspection (including the techniques used and how they are implemented).^c^	0 (0)		1 (4)		*26 (96)*	
	State whether a combination of techniques was used (eg, heuristic evaluation and cognitive walkthrough).	2 (7)		6 (22)		*19 (70)*	
	Provide the rationale for the choice of the techniques.	1 (4)		5 (19)		*21 (78)*	
	Detail the criteria to prioritize the resolution of problems identified (eg, according to the severity criteria, problems with higher impact on users are solved first).^a^	0 (0)		4 (15)		*23 (85)*	

^a^Values in italics denote the items that reached consensus on inclusion.

^b^New items that emerged from round 1.

^c^Items that were rephrased.

## Discussion

### Principal Results and Comparison With Previous Work

The results of this Delphi study are a set of agreed definitions of common terms used in the field of usability evaluation and a checklist of procedures that should guide the planning and reporting of usability evaluation studies. This consensus was achieved with a panel of international-experienced participants in usability evaluation, and these findings provide an important step toward a more standardized approach in the field of usability evaluation of digital health solutions for which the intended user is a layperson. We believe that the results of this study are particularly relevant for the evaluation of digital health solutions as they include terms and items that are specific to this field, such as the domain evaluator or details of the clinical conditions of the participants involved in the usability evaluation. Nevertheless, the general nature of most items suggests that the checklist is also relevant to inform usability evaluation in other fields.

Of the 38 items on procedures included in the Delphi, consensus on their relevance for the planning and reporting of usability studies was reached for 30. For the remaining 8, it was not possible to reach a consensus neither on their relevance nor on their irrelevance. These items were scored by more than 90% of participants with a rating of 4 or higher suggesting that they were considered moderately to highly relevant. Therefore, these items were also included in a checklist developed to facilitate the planning and reporting of usability studies (Multimedia Appendix 1). Nevertheless, results might suggest a division of the 38 items into 30 items that should be considered for all studies and 8 items that are important but not essential. Interestingly, the items for which consensus was not reached report mainly to aspects of the usability evaluation moderator (for evaluation involving users) and usability evaluator and domain evaluator (for evaluation involving experts), including the rationale for characteristics and sample size, inclusion criteria, and personal characteristics, which are seldom reported in the literature [19,32]. Conceivably, the individual characteristics of the person who conducts the usability evaluation and interacts with the participant guiding the session might impact the results as shown in previous studies [33-35]. The so-called “evaluator effect” is well known and has a great influence on the evaluation results as usability evaluation involves direct contact of the evaluator with the participant and a certain amount of subjective interpretation [33,34] that might impact results. For example, body language and tone of voice might influence how the user evaluates the digital solution [35]. Both the lack of reporting on existing literature and the percentage of participants classifying these as less relevant items might suggest a lack of awareness of the implications of the evaluator impact on the process of evaluation and, consequently, on its results.

A clear and distinctive characteristic of our checklist is its simplicity of use, objectivity, and high level of specification. For example, previous guidelines on evaluation in health informatics refer that users’ characteristics should be clearly identified and defined but do not identify a minimum set of user characteristics that need to be considered across studies allowing for different authors to report on different characteristics and making comparisons across studies difficult. Contrary, our checklist, exemplifies what information should be provided (eg, age, gender, educational level, digital literacy, and existing clinical conditions). We hope that this checklist can be a contribution toward a more standardized approach and high-quality planning and reporting of usability studies. This is likely to result in more robust studies and more transparent reports, which increase the interpretability and transferability of the results. In addition, it is likely to (1) increase comparability across studies and consequently, the aggregation of higher amounts of data into meta-analysis, (2) higher percentage of errors being detected during evaluations, (3) overarching studies comparing the level of sensitivity of different usability methods and techniques, to name a few potential gains for the field of usability.

We suggest that the list of terms and respective definitions should be used with the checklist to guarantee a common understanding. Furthermore, we acknowledge that the items of the checklist differ in the level of complexity and specificity. For example, while some refer to objectively stating what was done in the study (eg, “state whether the usability assessment moderators are external to service or product development team”), others report on the rationale of the decisions (eg, “provide a rationale to establish the sample size”), but we believe that this translates what is expected in the Methods section of a manuscript, where one needs to report both on methodological procedures and their justification. The checklist includes items that tend to be specific to usability studies, and important aspects such as data analysis, which is transversal to all studies, are not included. In these cases, authors should refer to existing guidelines and checklists, such as the Consolidated Criteria for Reporting Qualitative Research (COREQ) [36].

Given the lack of consensus for usability reporting, the process adopted in this Delphi method, which was conducted on the internet, was appropriate to assemble the views of an international panel of experienced participants on usability evaluation. In addition to identifying areas of consensus, this study was able to highlight areas where there is less certainty in the usability field, potentially requiring further research. One of the advantages of this method is that it allows participants to suggest new items that were not initially foreseen. Although the items sent to participants of the Delphi in the first round resulted from an extensive literature review [19,29], 9 new items were added to the initial list in the first round. Of the new items proposed by participants in the first round, the majority (7 out of 9) reached a consensus with only 1 round, which shows the adequacy and value of the Delphi method.

### Strengths and Limitations

The strength of the proposed checklist is that it was developed based on the perspective of an international panel of participants following a detailed analysis of existing evidence [27,28]. Being simultaneously a checklist to inform planning and reporting, it helps ensure that the important methodological aspects that need to be reported are also considered during the study planning. Although 30 participants are a number considered to be reasonable [27,28,37], it cannot be guaranteed that the views of the included participants are representative of the views of the broader community. In addition, Delphi participants were all from Europe, and most of them were from a small number of countries as half of the sample came from 2 countries (Portugal and Spain) potentially limiting the generalizability of the findings, particularly to those outside Europe. The participation rate cannot be calculated, as the invitation to participate in the Delphi study was spread across several European Projects of the Health and Care Cluster, Horizon 2020 Research and Innovation program, and there are no data on how many potential participants had access to the invitation to enter the Delphi. Furthermore, the inclusion criteria to be considered an experienced participant on usability were broad. Although they were selected among participants of European projects with a strong focus on usability and clarifications were made that participants had to be experienced on usability assessment, our inclusion criteria might not have been robust against the inclusion of individuals without an in-depth knowledge or experience of usability evaluation.

### Conclusions

This study proposes a set of terms and respective definitions and a checklist to guide the planning and reporting of usability evaluation studies both in the health area as well as for usability in general. These can be used both to guide the planning and reporting of usability evaluation studies as well as to inform quality assessment for these studies. Future studies can contribute to further validating this study work by refining the definitions, assessing the practical applicability of the current checklist for specific digital health solutions, or assessing whether using this checklist results in higher-quality digital health solutions.

## Appendix

Multimedia Appendix 1 Glossary and checklists of procedures for planning and reporting procedures for usability evaluation with users and experts.

## Abbreviations

ACTIVAGE: Activating Innovative IoT Smart Living Environments for Ageing Well
ADLIFE: Integrated Personalized Care for Patients With Advanced Chronic Diseases to Improve Health and Quality of Life
CONSORT: Consolidated Standards of Reporting Trials
COREQ: Consolidated Criteria for Reporting Qualitative Research
FAITH: Federated Artificial Intelligence Solution for Monitoring Mental Health Status After Cancer Treatment
InteropEHRate: Interoperable Electronic Health Records at user edge
SHAPES: Smart and Health Ageing through People Engaging in Supportive Systems

## Data Availability

The data sets generated and analyzed during this study are available from the corresponding author on request.

## References

[1] Martins AI, Queirós A, Rocha NP (2019). Validation of a usability assessment instrument according to the evaluators’ perspective about the users’ performance. Univ Access Inf Soc.

[2] Nielsen J (1993). Usability Engineering.

[3] (2019). Ergonomics of human-system interaction — part 210: human-centred design for interactive systems (ISO 9241-210:2019). International Organization for Standardization.

[4] Dix A, Finlay J, Abowd GD, Beale R (2004). Human-Computer Interaction, 3rd ed.

[5] Middleton B, Bloomrosen M, Dente MA, Hashmat B, Koppel R, Overhage JM, Payne TH, Rosenbloom ST, Weaver C, Zhang J (2013). Enhancing patient safety and quality of care by improving the usability of electronic health record systems: recommendations from AMIA. J Am Med Inform Assoc.

[6] Martins AI, Queirós A, Silva AG, Rocha NP, Bajwa IS, Saeed S, Mahmood Z (2015). Usability evaluation methods: a systematic review. Human Factors in Software Development and Design.

[7] Maguire M (2001). Methods to support human-centred design. Int J Hum Comput Stud.

[8] Petrie H, Bevan N, Stephanidis C (2009). The evaluation of accessibility, usability, and user experience. The Universal Access Handbook.

[9] (2013). Dispositivos médicos. Infarmed.

[10] European Union (1990). Council directive of 20 June 1990 on the approximation of the laws of the member states relating to active implantable medical devices (90/385/EEC). Off J Eur Comm.

[11] (2014). Human factors engineering for medical devices. Emergo UL.

[12] Kaye RD, North RA, Peterson MK (2003). UPCARE: an analysis, description, and educational tool for medical device use problems. https://citeseerx.ist.psu.edu/document?repid=rep1&type=pdf&doi=308668cf7912ca5ec10cefeabc0772f1feb3bcf8.

[13] Woods D, Cook RI (1999). The new look at error, safety, and failure: a primer for health care. Chicago National Patient Safety Foundation.

[14] Shaver E (2013). Human Factors and Medical Device Resources.

[15] Wiklund ME, Kendler J, Strochlic AY (2011). Usability Testing of Medical Devices. 1st ed.

[16] Bastien JMC (2010). Usability testing: a review of some methodological and technical aspects of the method. Int J Med Inform.

[17] Association for the Advancement of Medical Instrumentation (2013). Medical devices—application of usability engineering to medical devices ANSI/AAMI/IEC 62366:2007 (R2013). ANSI Webstore.

[18] Christ-Neumann M, Escrich A, Anguita A, Stenzhorn H, Taylor M, Ramay H, Rüping S, Krauth C, Kuchinke W, Graf N, Rossi S (2014). Usability on the p-medicine infrastructure: an extended usability concept. Ecancermedicalscience.

[19] Silva AG, Caravau H, Martins A, Almeida AMP, Silva T, Ribeiro O, Santinha G, Rocha NP (2021). Procedures of user-centered usability assessment for digital solutions: scoping review of reviews reporting on digital solutions relevant for older adults. JMIR Hum Factors.

[20] O'Connor SR, Kee F, Thompson DR, Cupples ME, Donnelly M, Heron N (2021). A review of the quality and content of mobile apps to support lifestyle modifications following a transient ischaemic attack or 'minor' stroke. Digit Health.

[21] Regmi D, Tobutt C, Shaban S (2018). Quality and use of free smoking cessation apps for smartphones. Int J Technol Assess Health Care.

[22] Almeida AF, Rocha NP, Silva AG (2020). Methodological quality of manuscripts reporting on the usability of mobile applications for pain assessment and management: a systematic review. Int J Environ Res Public Health.

[23] Nykänen P, Brender J, Talmon J, de Keizer N, Rigby M, Beuscart-Zephir M, Ammenwerth E (2011). Guideline for good evaluation practice in health informatics (GEP-HI). Int J Med Inform.

[24] Bevan N (1999). Common industry format usability tests. Proceedings of Usability Professionals Association.

[25] Nouri R, R Niakan Kalhori S, Ghazisaeedi M, Marchand G, Yasini M (2018). Criteria for assessing the quality of mhealth apps: a systematic review. J Am Med Inform Assoc.

[26] CONSORT Group CONSORT—Consolidated Standards of Reporting Trials.

[27] Hasson F, Keeney S, McKenna H (2000). Research guidelines for the Delphi survey technique. J Adv Nurs.

[28] Hsu CC, Sandford BA (2007). The Delphi technique: making sense of consensus. Pract Assessment Res Eval.

[29] Silva A, Isabel Martins A, Caravau H, Martins Almeida A, Silva T, Ribeiro O, Santinha G, Rocha N (2020). Experts evaluation of usability for digital solutions directed at older adults: a scoping review of review. https://dl.acm.org/doi/abs/10.1145/3439231.3439238.

[30] Veugelers R, Gaakeer MI, Patka P, Huijsman R (2020). Improving design choices in Delphi studies in medicine: the case of an exemplary physician multi-round panel study with 100% response. BMC Med Res Methodol.

[31] Thorn JC, Brookes ST, Ridyard C, Riley R, Hughes DA, Wordsworth S, Noble SM, Thornton G, Hollingworth W (2018). Core items for a standardized resource use measure: expert Delphi consensus survey. Value Health.

[32] Ellsworth MA, Dziadzko M, O'Horo JC, Farrell AM, Zhang J, Herasevich V (2017). An appraisal of published usability evaluations of electronic health records via systematic review. J Am Med Inform Assoc.

[33] Hertzum M, Jacobsen NE (2003). The evaluator effect: a chilling fact about usability evaluation methods. Int J Hum-Comput Interact.

[34] Hertzum M, Molich R, Jacobsen NE (2014). What you get is what you see: revisiting the evaluator effect in usability tests. Behav Inf Technol.

[35] Riihiaho S, Kirakowski J, Norman K (2018). Experiences with usability testing: effects of thinking aloud and moderator presence. The Wiley Handbook of Human Computer Interaction. Vol 1.

[36] Tong A, Sainsbury P, Craig J (2007). Consolidated criteria for reporting qualitative research (COREQ): a 32-item checklist for interviews and focus groups. Int J Qual Health Care.

[37] Murphy MK, Black NA, Lamping DL, McKee CM, Sanderson CF, Askham J, Marteau T (1998). Consensus development methods, and their use in clinical guideline development. Health Technol Assess.

