# Supplementary material for: Consensus on the Terms and Procedures for Planning and Reporting a Usability Evaluation of Health-Related Digital Solutions: Delphi Study and a Resulting Checklist
Source: J Med Internet Res. 2023 Jun 6;25:e44326. doi: 10.2196/44326 (PMC10282913; doi:10.2196/44326)
Supplement: Multimedia Appendix 1 [file jmir_v25i1e44326_app1.pdf]

I. Glossary	
**Term**	**Definition**
Usability assessment moderator	The person who conducts the usability evaluation, interacts with the participant, and guides the session.
Participant	The person who is asked to evaluate the usability of a product or service and who completes the tasks, following the indications of the evaluator.
Usability evaluation method	A set of techniques used to perform usability evaluation at different stages of the product or service development (example: inquiry or test methods).
Tasks	Self-contain/independent activities that participants are asked to perform when evaluating the usability of a product or service within a limited period of time.
Usability evaluation technique	A set of procedures used to perform a usability evaluation and collect either qualitative or quantitative data (example: focus group, survey, think aloud and performance).
Usability evaluation environment	The environment where the evaluation of usability takes place: (i) laboratory or controlled conditions, (ii) in a real context, i.e., the usability evaluation is carried out in the same context and circumstances where the end product is expected to be used.
Usability evaluator	Person with knowledge and experience on Human-Computer Interaction (HCI), usability and user experience that conducts the usability inspection (e.g., an HCI specialist assigned to evaluate the interface of a technology for patients with diabetes).
Domain evaluator	Person with knowledge on the application area of the technology under development that provides feedback about functionalities of the technologic product or service (e.g., a physician involved in treating patients with diabetes who provides feedback about technology for patients with diabetes).

II. Checklist of procedures for planning and reporting procedures for usability evaluation with users

**Subject**	**Item no**	**Checklist item**	**Reported on page No**
Usability assessment moderator	1a	Specify as inclusion criteria having previous experience with usability evaluation with users or consider adequate training and provide details of the training plan.	______
	1b	Specify whether the usability assessment moderator(s) is external to the service or product development team.	______

	1c	Specify if observers are included, define their responsibilities, and collect their characteristics (e.g., gender, academic background, and previous experience in usability evaluation).
Participants	2a	Determine sample size (i.e., the total number of participants involved in the evaluation).
	2b	Provide a rationale to establish the sample size.
	2c	Provide clear inclusion and exclusion criteria (e.g., profile definition including age, gender, educational level, digital literacy, previous experience using the product or service being evaluated).
	2d	Provide sampling methods (e.g., random, systematic, cluster, convenience, snowball).
	2e	Indicate the setting of participants' recruitment (e.g., community, hospital).
	2f	Detail clinical conditions (if relevant for the study): (e.g., asymptomatic or with a specific clinical condition or from a specific group - occupational group, the severity of the clinical condition, disabilities, cognitive impairment).
	2g	Detail the participant's characteristics that should be collected (such as age, gender, educational level, digital literacy).
Usability evaluation method and usability evaluation technique	3a	Specify whether a combination of usability evaluation methods is used (e.g., using both inquiry and test methods).
	3b	Specify whether a combination of usability evaluation techniques is used (e.g., for the inquiry method combine the questionnaire and interview techniques).
	3c	Provide the rationale for the choice of usability evaluation method(s) and technique(s).
	3d	Describe the usability evaluation method(s) and technique(s) used and how they are implemented.
	3e	When using measuring instruments such as scales or questionnaires, give indicators of their validity and reliability.
	3f	Describe the data analysis plan for both quantitative and qualitative data.
Tasks	4a	Provide a detailed description of tasks or present the session script.
	4b	Indicate the total number of tasks.
	4c	Detail the tasks related outcomes and how they are measured (e.g., task completion and duration, and a number of errors).
	4d	Detail the conditions for carrying out the tasks (e.g., with or without supervision, individually or in a group, with or without a period for familiarization with the digital product or service).
	4e	Detail the instructions to participants and the way they are presented (e.g., verbally; written, both) and registered (e.g., audio, video, screen recorder, notes from an observer).
Usability evaluation environment	5a	Justify the choice of the usability evaluation environment (e.g., lab or field test; remote or face-to-face test).
	5b	Specify usability evaluation environment requirements (e.g., recording equipment or observer room

		availability).	
**III. Checklist of procedures for planning and reporting procedures for usability evaluation with experts**			
**Subject**	**Item no**	**Checklist item**	**Reported on page No**
For the usability evaluator and the domain evaluator	1a	Specify as inclusion criteria having previous experience in inspection usability evaluation or consider adequate training and provide details of training	______
	1b	State whether a combination of evaluators from different domains is used (e.g., for a health-related digital service, use both usability and health domain evaluators).	______
	1c	Provide clear inclusion and exclusion criteria.	______
For the inspection method	2a	Detail the protocol to conduct the inspection (including the technique(s) used and how they are implemented).	______
	2b	State whether a combination of techniques is used (e.g., heuristic evaluation and cognitive walkthrough)	______
	2c	Provide the rationale for the choice of the technique(s)	______
	2d	Detail the criteria to prioritize the resolution of problems identified (e.g., according to the severity criteria, problems with higher impact on users are solved first).	______

This is a Multimedia Appendix to the full manuscript "Consensus on the Terms and Procedures for Planning and Reporting Usability Evaluation of Health-Related Digital Solutions: Delphi Study and a Resulting Checklist", published in the J Med Internet Res.

For full copyright and citation information see http://dx.doi.org/10.2196/jmir.44326
